# A simple magnetoencephalographic auditory paradigm may aid in confirming left-hemispheric language dominance in epilepsy patients

**DOI:** 10.1371/journal.pone.0200073

**Published:** 2018-07-02

**Authors:** Juha Wilenius, Henri Lehtinen, Ritva Paetau, Riitta Salmelin, Erika Kirveskari

**Affiliations:** 1 Clinical Neurosciences, Department of Clinical Neurophysiology, HUS Medical Imaging Center, University of Helsinki and Helsinki University Hospital, Helsinki, Finland; 2 Epilepsy Unit, Department of Pediatric Neurology, University of Helsinki and Helsinki University Hospital, Helsinki, Finland; 3 Department of Neuroscience and Biomedical Engineering, Aalto University, Espoo, Finland; Australian Research Council Centre of Excellence in Cognition and its Disorders, AUSTRALIA

## Abstract

**Objective:**

The intracarotid amobarbital procedure (IAP) is the current “gold standard” in the preoperative assessment of language lateralization in epilepsy surgery candidates. It is, however, invasive and has several limitations. Here we tested a simple noninvasive language lateralization test performed with magnetoencephalography (MEG).

**Methods:**

We recorded auditory MEG responses to pairs of vowels and pure tones in 16 epilepsy surgery candidates who had undergone IAP. For each individual, we selected the pair of planar gradiometer sensors with the strongest N100m response to vowels in each hemisphere and—from the vector sum of signals of this gradiometer pair—calculated the vowel/tone amplitude ratio in the left (L) and right (R) hemisphere and, subsequently, the laterality index: LI = (L–R)/(L+R). In addition to the analysis using a single sensor pair, an alternative analysis was performed using averaged responses over 18 temporal sensor pairs in both hemispheres.

**Results:**

The laterality index did not correlate significantly with the lateralization data obtained from the IAP. However, an MEG pattern of stronger responses to vowels than tones in the left hemisphere and stronger responses to tones than vowels in the right hemisphere was associated with left-hemispheric language dominance in the IAP in all the six patients who showed this pattern. This results in a specificity of 100% and a sensitivity of 67% of this MEG pattern in predicting left-hemispheric language dominance (p = 0.01, Fisher's exact test). In the analysis using averaged responses over temporal channels, one additional patient who was left-dominant in IAP showed this particular MEG pattern, increasing the sensitivity to 78% (p = 0.003).

**Significance:**

This simple MEG paradigm shows promise in feasibly and noninvasively confirming left-hemispheric language dominance in epilepsy surgery candidates. It may aid in reducing the need for the IAP, if the results are confirmed in larger patient samples.

## Introduction

Resective brain surgery is a feasible treatment option in many epilepsy patients whose disease is intractable to medication—for a recent review, see[1]. Comprehensive preoperative examinations are performed to select those patients who probably benefit from surgery, and to define the location and extent of the cortical resection. The aim of preoperative evaluation is to maximize the positive treatment effects while minimizing the risk of any unacceptable loss of function caused by the operation.

Epilepsy patients have a high incidence of atypical–that is, bilateral or right dominant–language representation; for a review, see[2]. Therefore, assessing the hemispheric lateralization of language functions is an essential part of the presurgical examination in those patients whose suspected epileptogenic zone is in the vicinity of eloquent language-related areas.

The intracarotid amobarbital procedure (IAP), also known as the Wada test, where a short-acting barbiturate is used to anesthetize one hemisphere at a time, has been the “gold standard” test for assessing the hemispheric lateralization of language[3]. It is invasive and its results are often difficult to interpret because of various limitations such as limited duration of language testing due to typically short effect of the anesthetic, drug cross-over to the other hemisphere and the general sedative effect of the drug. Complication rates of up to 11% have been reported[4]. Therefore, it should be replaced by non-invasive alternatives whenever sufficient and available.

In addition to the IAP, traditional invasive methods utilized in language mapping are direct cortical stimulation during awake craniotomy[5] and stimulation by intracranial EEG electrodes[6]. These methods are highly invasive and also have limitations, in particular the limited coverage of cortex.

Noninvasive functional neuroimaging methods such as functional magnetic resonance imaging (fMRI), navigated transcranial magnetic stimulation (nTMS) and magnetoencephalography (MEG) have been utilized to localize language-related cortical areas. Of these methods, fMRI is currently the most established noninvasive method for language lateralization in epilepsy patients. Some recent reviews have concluded that fMRI is a reliable method in confirming left-hemispheric language dominance, but when it fails to show this typical dominance, IAP may still be needed[7,8]. Another review of language fMRI in healthy adults pointed out that some problematic arbitrary decisions must be made during the analysis, for example thresholding and definition of region-of-interest (ROI)[9]. Although the different methods utilized in language fMRI studies make systematic reviews difficult, substantial scientific evidence has been accumulated of its utility in studying the language lateralization in neurosurgical patients. Therefore, despite its limitations, language fMRI has become the new “gold standard” in many epilepsy surgery centers where the IAP is utilized rarely or not at all.

Language mapping with nTMS is a novel tool that has shown good correlation with direct cortical stimulation in brain tumor patients[10,11]. However, the lateralizing value of nTMS in language mapping appears limited and it might be lower in neurological patients than in healthy subjects[12].

Previous studies comparing the assessment of language lateralization performed with MEG *vs*. the IAP have generally shown good concordance. For example, Papanicolaou et al.[13] reported a concordance of 87% in a series of 100 epilepsy patients using a word recognition task in MEG. However, 15 of the original 100 patients had to be excluded from the analysis because of inadequate quality of the evoked MEG responses. A later study replicated the methodology used by Papanicolaou et al. in 35 patients and reported a somewhat lower concordance rate of 69%[14]. In those studies, the laterality index (LI) was calculated based on the number of neural sources modeled as equivalent current dipoles that met certain criteria in each hemisphere. Despite good concordance with the IAP, from a clinical perspective the shortcoming of the protocol used in these studies is the time consuming and somewhat subjective analysis.

Other MEG studies of language lateralization have utilized various analysis methods and language tasks: dynamic statistical parameter mapping (dSPM) with a semantic word processing task[15] and analysis of oscillatory responses by spatial filtering using a silent reading task[16] or a verb generation task[17]. These paradigms require processing the patients’ individual MR images to construct source space and conductor models, and to define the ROI for LI calculations. The authors have reported high concordance rates with the IAP ranging from 85% to 93%.

Compared to fMRI, MEG is not yet well-established as a clinical tool for language lateralization, and therefore further studies of its use for this purpose are warranted. It would always be preferable to have language lateralization results from several independent noninvasive methods–such as fMRI and MEG–available for clinical decision making.

MEG is utilized to localize the sources of epileptic activity in many epilepsy surgery candidates, especially in patients with no lesions apparent in the MRI[18,19]. It would be beneficial if during the very same MEG session, language lateralization could be assessed with a test that can be added to the recording and analyzed with minimal added effort. Therefore, we decided to investigate the usefulness of a simple auditory paradigm with sensor level analysis in the assessment of language lateralization in epilepsy surgery candidates. The analysis is semi-automated and fast to perform with minimal input from the MEG operator. This paradigm was previously found to elicit differently lateralized N100m responses to vowels *vs*. pure tones in healthy right-handed subjects compared to left-handed subjects[20].

## Methods

### Patients

The study group included 16 epilepsy patients (9 males) who had undergone MEG and the IAP for determination of hemispheric language lateralization as a part of their preoperative assessment for epilepsy surgery. The results of our MEG language lateralization test did not influence the clinical decision making during the presurgical evaluations. Children under the age of 12 years were excluded from the study because of developmental inconsistency of the N100m response[21]. Fifteen patients were native Finnish speakers and one patient a native English speaker with good Finnish skills. Information on the handedness was obtained from the patients' medical history. Table 1 includes a detailed clinical description of the patients.

**Table 1:** Clinical details of the patients.

Patient	Sex	Age	Handedness	Epileptic focus	Seizure type(s)	Etiology	Duration of epilepsy (yrs)	Antiepileptic drugs
1	M	47	Right	Left temporal mesial	Aura → Dyscognitive→ GTC	Encephalocele, mesial temporal sclerosis	24	TPM, CZP
2	M	38	Right	Left temporal mesial	Dyscognitive → GTC	Mesial temporal sclerosis	8	LTG, OXC, PGB, CLB
3	F	30	Right	Left temporal basal	Aura → Dyscognitive → GTC	Glioma gr I	26	LTG, LEV
4	F	43	Right	Left temporal posterior basal	Dyscognitive → GTC	Glioma gr I	15	LEV, PGB, CBZ
5	M	43	Right	Left temporal	Aura → Dyscognitive → GTC	Unknown	5	PGB, OXC
6	M	37	Left	Right temporal	Aura → GTC	HSV-encephalitis	9	VPA, LTG
7	M	27	Right	Bilateral temporal	Aura → Dyscognitive → GTC	Unknown	7	PGB, LEV
8	F	50	Right	Bilateral temporal	Aura → Dyscognitive → GTC, post-ictal psychosis	Mesial temporal sclerosis (left)	47	CBZ, CLB, DZP
9	M	15	Right	Left temporo-parietal	Aura → Dyscognitive → GTC, (previously spasms)	Unknown	14	OXC, LCM
10	F	21	Right	Left temporal posterior lateral	Aura → Dyscognitive → GTC	FCD Ia (MRI-negative)	11	OXC, PGB, CLB
11	F	47	Right	Left temporal anterior	Aura → Dyscognitive → GTC	FCD Ia (MRI-negative)	12	LEV
12	M	17	Left	Left temporo-parieto-occipital	Clonic (right), Dyscognitive → GTC	HSV-encephalitis	17	OXC, CLB
13	M	16	Right	Right frontal	Spasms, atypical absence, GTC	FCD Ia	6	VPA, LTG, CLB
14	F	24	Right	Left temporal anterior	Aura → Dyscognitive → GTC	FCD Ib (MRI-negative)	12	CBZ, VPA, PGB
15	F	16	Right	Left temporal mesial	Aura -> Dyscognitive	Mesial temporal sclerosis	2	CBZ, PGB
16	M	17	Left	Left parieto-insular	Right sensory, right tonic-clonic, bilateral tonic, GTC	Perinatal infarction	12	OXC, VPA. ZNS

Seizure semiology is described according to the local convention (based on the Cleveland Clinic terminology); the corresponding terms in ILAE 2017 classification are: Aura: focal seizure with preserved awareness, Dyscognitive: Focal seizure with impaired awareness. Arrows describe the temporal evolution of the seizure.

Abbreviations: GTC: generalized tonic-clonic; FCD: focal cortical dysplasia; HSV: herpes simplex virus; CBZ: carbamazepine; PGB: pregabalin; CLB: clobazam; LEV: levetiracetam; LTG: lamotrigine; OXC: oxcarbazepine; TPM: topiramate; VPA: valproic acid; LCM: lacosamide; ZNS: zonisamide; CZP: clonazepam; DZP: diazepam.

Twelve of the patients had resective surgery after the MEG recording. Seven patients underwent left temporal lobe resection. One patient had an extensive resection of the posterior areas of the left hemisphere. Resection of left parieto-insular region, left temporo-parietal region, right temporal lobe and right frontal lobe were performed in one patient each. Additionally, one patient had undergone previous resective epilepsy surgery, but has not had a re-operation after the MEG recording.

For the patients who were operated, we reviewed the postoperative neuropsychological examinations and categorized any postoperative language deficits not present before the operation as none (-), mild (+), substantial (++) or severe (+++), see Table 2. Five youngest patients followed a pediatric protocol with two postoperative examinations at two and six months after surgery. Basic language functions and verbal memory were tested in the first evaluation, and verbal intelligence in the second evaluation. The rest of the patients followed an adult protocol with only one thorough postoperative neuropsychological examination at six months after the surgery.

**Table 2:** Results of language studies.

Patient	Handedness	IAP	MEG LI (single sensor)	MEG LI (regional average)	MEG “LH pattern”	fMRI	nTMS	Intracranial stimulations	Post-operative language deficit
1	Right	Bilateral^a^	-0.10	-0.12	-	-	-	-	++
2	Right	Left	-0.20	-0.18	-	-	-	-	++
3	Right	Left	0.29	0.31	+	-	-	-	-
4	Right	Left	-0.01	-0.06	-	Inconclusive	-	-	Not operated
5	Right	Bilateral^a^	0.24	0.29	-	-	-	-	Not operated
6	Left	Left^b^	0.30	0.29	+	Left	-	-	-
7	Right	Left	0.44	0.45	+	Left	-	-	Not operated
8	Right	Left^b^	-0.04	0.09	(+)^f^	-	-	-	Not operated
9	Right	Bilateral	-0.12	0.04	-	-	Bilateral	Grid: left side implantation, language response +	+
10	Right	Bilateral	-0.14	-0.14	-	-	Left (right not stimulated)	Grid: left side implantation, language response +	-
11	Right	Bilateral^a^	0.16	0.11	-	-	-	SEEG: left side implantation, language response +	+
12	Left	Right/bilateral^c^	0.12	0.34	-	Right or bilateral	-	SEEG: left side implantation, language response +	-/improvement
13	Right	Left^b^	0.16	0.22	+	-	-	-	-
14	Right	Left	0.32	0.32	+	Left	-	SEEG: left side implantation, language response +	-
15	Right	Left^b^	0.12	0.07	+	-	Bilateral	-	+
16	Left	Right/bilateral^d^	-^e^	-^e^	-	-	Left (right not stimulated)	Grid: left side implantation, language response -	-

a: limited right hemisphere language function

b: bilateral language representation not conclusively ruled out

c: only left hemisphere injection was performed

d: patient was unresponsive to testing during right hemisphere injection

e: no response to tone stimuli in the left hemisphere

f: “LH pattern” when analysis was based on responses averaged over temporal channels

Post-operative language deficit: -: no deficit, +: mild deficit, ++: substantial deficit.

Abbreviations: IAP: intracarotid amobarbital procedure; MEG: magnetoencephalography; LI: laterality index; LH: left hemisphere; fMRI: functional magnetic resonance imaging; nTMS: navigated transcranial magnetic stimulation; SEEG: stereotactic electroencephalography.

Six patients underwent intracranial EEG studies (subdural grid electrodes or stereotactic EEG) with stimulation of the cortical areas related to language function during a picture naming task. In all of these patients, electrode implantation was limited to the left hemisphere. An additional patient had intracranial electrodes implanted, but as they were limited to the anterior temporal structures bilaterally, no language related stimulations were performed.

The study had a prior approval by the Ethics Committee of the Hospital District of Helsinki and Uusimaa (number 459/13/03/03/2008). A written informed consent was obtained from the patients and additionally from a parent or a guardian of the patients less than 18 years old.

### Intracarotid amobarbital procedure (IAP)

All IAPs were done with sodium amobarbital as the sedative agent. To practice for the actual procedure and to establish the baseline performance, at least one training run of language and memory testing was done at least one day prior to the actual procedure. During the IAP, both hemispheres were tested starting with anesthetizing the side of suspected seizure onset. There was a minimum time period of 30 minutes between the sodium amobarbital injections to each hemisphere. In one patient only the left hemisphere was anesthetized, as that yielded all necessary information to continue with surgical treatment.

The localization, intensity and duration of sodium amobarbital effect was judged by the results of cerebral angiography, EEG recording during testing, judgment of hemiparesis from bimanual hand squeezing during the test as well as by other behavioral effects observed. Digital video and audio were recorded for offline analysis.

A single bolus injection was always used, the dose being typically 125 mg. In two patients, the dose was 150 mg. For one patient, an initial dose of 125 mg failed to give a satisfactory response on EEG or clinically on either side, so a new dose of 175 mg was used on both sides. One patient showed excessive cross-flow between hemispheres in angiography resulting in bilateral effects of sodium amobarbital and occasional non-habitual seizures. In this patient, doses of 125, 150 and 175 mg were all injected into the left hemisphere during two separate sessions aiming for a good clinical response, but with no success.

Due to short-lasting effect of sodium amobarbital, the testing protocol varied depending on whether the primary indication for the IAP was to assess only language, only episodic memory or both. In 11 patients, episodic memory assessment was crucial. Our previous IAP memory protocol with a relatively restricted set of language tests was used in 10 of these patients. The language tests included naming objects, reading words and understanding the verbal instruction to squeeze hands. In addition, single requests to state one’s name and to repeat a single word were presented at the beginning of the test shortly after the injection. In one patient, our current IAP memory protocol with better balance between memory and language testing was used. Language testing in this case included naming, concrete and abstract understanding, serial speech, repetition, reading, spelling and synonym generation with the option of complying without expressive speech in some of these tasks. In the remaining five patients, language could be tested most comprehensively as testing the episodic memory function was not relevant for the surgical planning. An individually tailored set of tasks always covering both expressive and receptive language function was used, including for example naming, reading, repetition, serial speech, simple arithmetics, answering simple semantic questions and following simple instructions with varying amount of semantic and concrete content. It was always possible to perform some of the tasks without expressive speech.

The results for hemispheric lateralization of language were categorized as: (1) left dominant, with no or very limited language functions during left-sided injection and full language function during right-sided injection; (2) probably left dominant, in the patients with full language function after right-sided injection but incomplete testing after left-sided injection; (3) bilateral with left dominance, with limited language functions during left-sided injection; (4) bilateral, with full or almost full language functions during left and right-sided injections; and (5) right dominant/bilateral, in the patients with full language function after left-sided injection but who had incomplete testing after right-sided injection or only left-sided injection was performed.

### MEG recordings and stimuli

MEG was recorded with a 306-channel neuromagnetometer (Vectorview^TM^, Elekta Oy, Helsinki, Finland), which has two orthogonal planar gradiometers and one magnetometer at each of the 102 sensor locations. The signals were sampled at 600 Hz except for one patient, whose recording was performed with a sampling frequency of 1000 Hz. Four head position indicator coils were attached to the patient's head. The locations of these coils and three anatomical landmarks (nasion, left and right preauricular points) were registered in order to assess the head position in relation to the MEG device at the beginning of each recording session. In seven patients, head position was also continuously measured throughout the recording. An electro-oculogram (EOG) was recorded in 14 patients.

Patients were presented with auditory stimuli comprised of pairs of vowels and pairs of tones via a panel speaker located in front of the subject at the midline. The sound level was adjusted for each patient to achieve clearly audible yet comfortable stimulus volume. The order of these two stimulus types was randomized for each session. The vowel stimuli included the Finnish vowels /a/, /e/, /i/, /o/ and /u/. The tone stimuli were pure tones with frequencies of 394, 442, 496, 525 and 589 Hz. The duration of a single vowel or tone was 250 ms and the silent interval between the two stimuli in a pair was 70 ms. The inter-stimulus interval was 2520 ms and thus the silent interval between two pairs of stimuli was 1950 ms. To control vigilance, patients were instructed to respond with lifting the index finger of their non-dominant hand to pairs of identical vowels or identical tones (20% of all stimuli). Averaged MEG responses to the non-target pairs of different vowels and different tones were used for analysis.

### MEG data analysis

Data were processed using the SSS artefact removal software Maxfilter by Elekta Oy[22],and in patients with continuous head position tracking, movement compensation was utilized (default settings were used). In six patients the data were of inadequate quality even after SSS; in those cases the data were re-processed using SSS with temporal extension (tSSS) (correlation limit 0.98, buffer length 16 seconds). Five patients had their MEG data in two separate data files recorded during the same MEG session. In these patients the head position in the second data file was transferred using SSS/tSSS to match the head position in the first data file in order to combine the data in these files. The data were averaged offline and artefact rejection (e.g. for epileptic discharges) was performed based on visual inspection of the signal. An electro-oculogram (EOG) based rejection of epochs contaminated with blinks or saccades was also utilized using a rejection limit of 150 μV. In each patient, we averaged a minimum of 100 artefact-free responses in both stimulus categories. The averaged epoch started at 200 ms before stimulus and ended at 800 ms after stimulus. Baseline was defined to start at 190 ms before stimulus and to end at 10 ms before the stimulus.

The auditory evoked responses were low-pass filtered at 40 Hz (zero-phase FIR filter, passband ripple of ±0.015 dB, stopband attenuation of -60 dB, transition band from 40 to 80 Hz) and baseline correction to remove the DC offset was applied. Planar gradiometer data were chosen for analysis because this type of an MEG sensor shows maximal signal directly over the location of the source current underneath (assuming a homogenous spherical conductor) and is therefore well suited for sensor level analysis[23]. Gradiometers are also generally less prone to external noise than magnetometers. A vector sum was calculated for each pair of orthogonal gradiometers in order to quantify the strength of the response irrespective of the magnetic field’s orientation ($vectorsum=\sqrt{{\left(\frac{\partial B_{z}}{\partial x}\right)}^{2}+{\left(\frac{\partial B_{z}}{\partial y}\right)}^{2}}$, where $\frac{\partial B_{z}}{\partial x}$ and $\frac{\partial B_{z}}{\partial y}$ are the outputs of the two orthogonal gradiometers at one sensor location).

Following the procedure described in Gootjes et al.[24], the sensor site (pair of gradiometers) with the strongest N100m response to vowel stimuli over each temporal region was selected for each individual, and the vowel/tone amplitude ratio in the left (L) and right (R) hemisphere was quantified using the average values of the vector sums between -25 ms and +25 ms with respect to the peak latency. The laterality index (LI) was then calculated as: $LI=\frac{L-R}{L+R}$, with positive LI values suggesting left-hemispheric language dominance.

As an additional measure, a regional laterality index (LI-regional) was calculated using averaged vector sums of responses over a set of 18 temporal sensor pairs in each hemisphere, instead of a single planar gradiometer pair per hemisphere. The selection of sensors was based on the visual analysis of the distributions of the N100m responses over all patients. The same sensor selection covered well the field pattern of the auditory response in all patients.

After SSS/tSSS and visual inspection of the data, the rest of the analysis described above was performed automatically using a custom built MATLAB script utilizing the MNE MATLAB toolbox[25]. The responses from which the laterality index was calculated were also visually checked to ensure good data quality.

For the patients who displayed the “left hemisphere language dominant MEG pattern” (defined in Results), we performed additional *post hoc* analysis to assess whether the observed pattern was truly distinct. For each patient, we normalized the amplitudes of the MEG responses by scaling them such that the peak amplitude of the N100m response to the vowel stimuli in the left hemisphere and to the tone stimuli in the right hemisphere were set to the value of one. The response to the other stimulus category within each hemisphere was scaled with respect to this selected reference response to maintain the relative amplitudes of the responses to tones and vowels. The responses of the individual patients were also shifted in time to align the peak latencies of the N100m responses to both stimulus types in both hemispheres. Grand average responses were then calculated and the mean relative amplitudes of the N100m responses to the tone stimuli in the left hemisphere and to the vowel stimuli in the right hemisphere were determined including their 95% confidence intervals. This analysis was performed similarly for both the single gradiometer pair and the regional responses.

### Statistics

Fisher's exact test was used to statistically assess the association between a certain MEG response pattern (the “left hemisphere language dominant MEG pattern”, defined in Results) and left-dominant IAP result. Spearman rank correlation was utilized to test whether there was a correlation between different IAP result categories and MEG laterality index. Wilcoxon rank sum test was used to test the difference between MEG laterality indices of left-dominant (based on the IAP) and non-left-dominant patients. In the *post hoc* analysis of the patients with the “left hemisphere language dominant MEG pattern”, the 95% confidence intervals of amplitude were calculated utilizing Student's t distribution with degrees of freedom of n-1, where n is the number of patients included in the analysis.

## Results

### Intracarotid amobarbital procedure (IAP)

Nine of the sixteen patients showed left-hemispheric language dominance (Table 2). In four of these patients, full language function was confirmed after right-sided injection, but left-sided injection caused either total sedation, fluctuating agitation, a state resembling akinetic mutism or seizures obliterating testing efforts. Thus, bilateral language representation could not be completely ruled out in these cases, but left-hemispheric dominance was considered probable because none of these patients had any other findings suggesting bilateral language representation or right-hemispheric dominance other than one patient being left-handed. The epileptic focus was located in the left hemisphere in all of these four patients.

Five patients were found to have bilateral language representation: three patients had limited language functions during left-sided injection and two patients had full or almost full language functions during both left and right-sided injections.

Two patients had full language function during left-sided injection and were considered right-dominant. However, bilateral language functions could not be ruled out in these two patients because in one of them only left-sided injection was performed and the other patient was unresponsive to testing during the right-sided injection due to an akinetic mutism–like state. Both of these patients were left-handed.

### Magnetoencephalography (MEG)

All but one patient showed N100m responses in both hemispheres to both vowel and tone stimuli. One patient lacked the N100m response to tone stimuli in the left hemisphere and, therefore, the laterality index could not be calculated for this patient.

The mean peak latency (± SD) of the N100m response in the left hemisphere was 122 (± 23) ms for the vowel stimuli and 124 (± 21) ms for the tone stimuli in the single sensor analysis. In the right hemisphere, mean peak latency was 120 (± 29) ms for vowels and 121 (± 30) ms for tones. When averaged responses over the temporal sensors were used for analysis, the mean peak latency was 126 (± 22) ms (vowels) and 121 (± 20) ms (tones) in the left hemisphere and 119 (± 29) ms (vowels) and 128 (± 26) ms (tones) in the right hemisphere.

The patients detected on average 83% of the target stimuli. Ten patients had a detection rate of more than 90%. Two patients detected less than 50% of the target stimuli (41% and 5%). In one patient, the target stimuli detection data were not present in the MEG recording.

**Fig 1:**
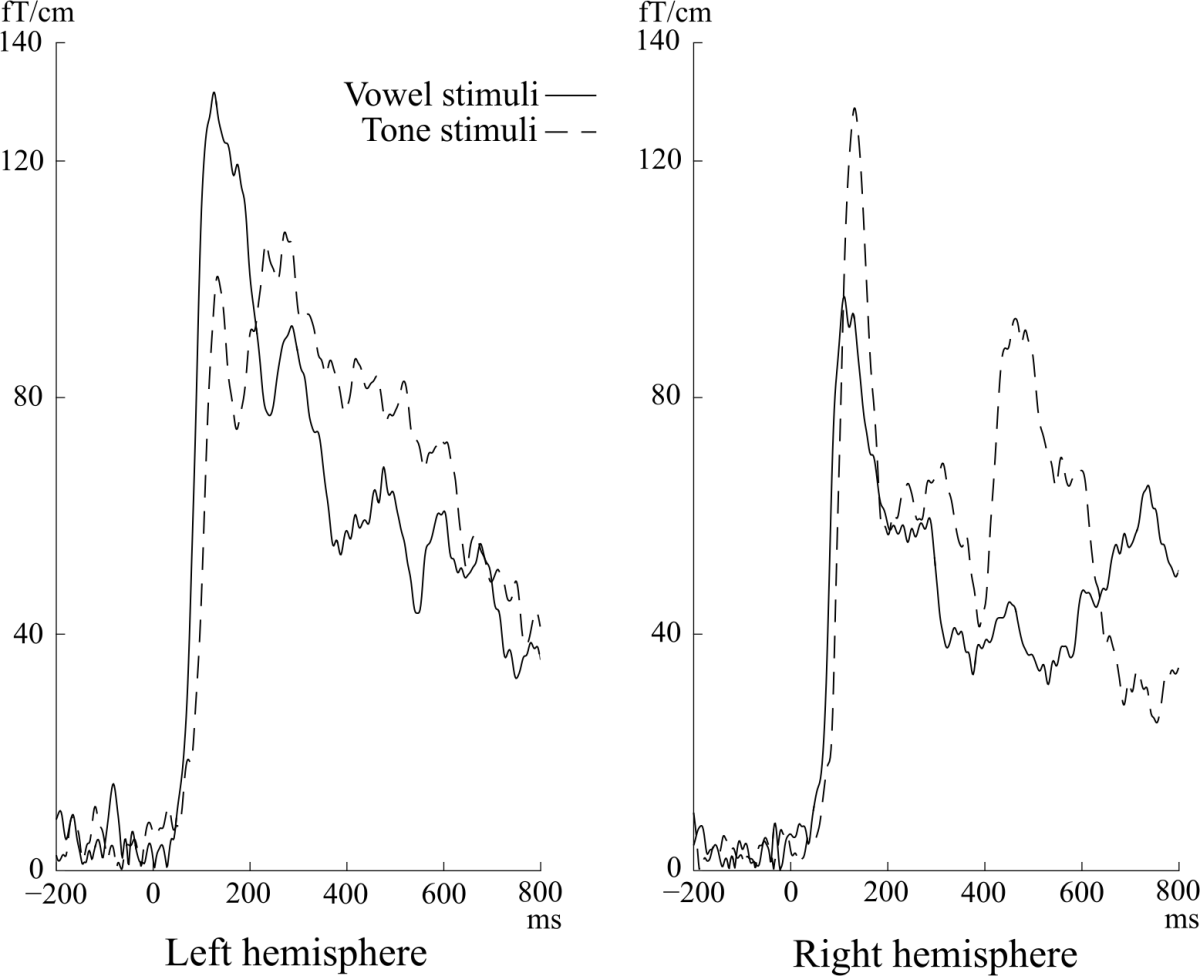
An example of the “left hemisphere language dominant MEG pattern”. MEG responses of one patient (P3). The N100m response to the vowel *vs*. tone stimuli is stronger in the left hemisphere and *vice versa* in the right hemisphere.

**Fig 2:**
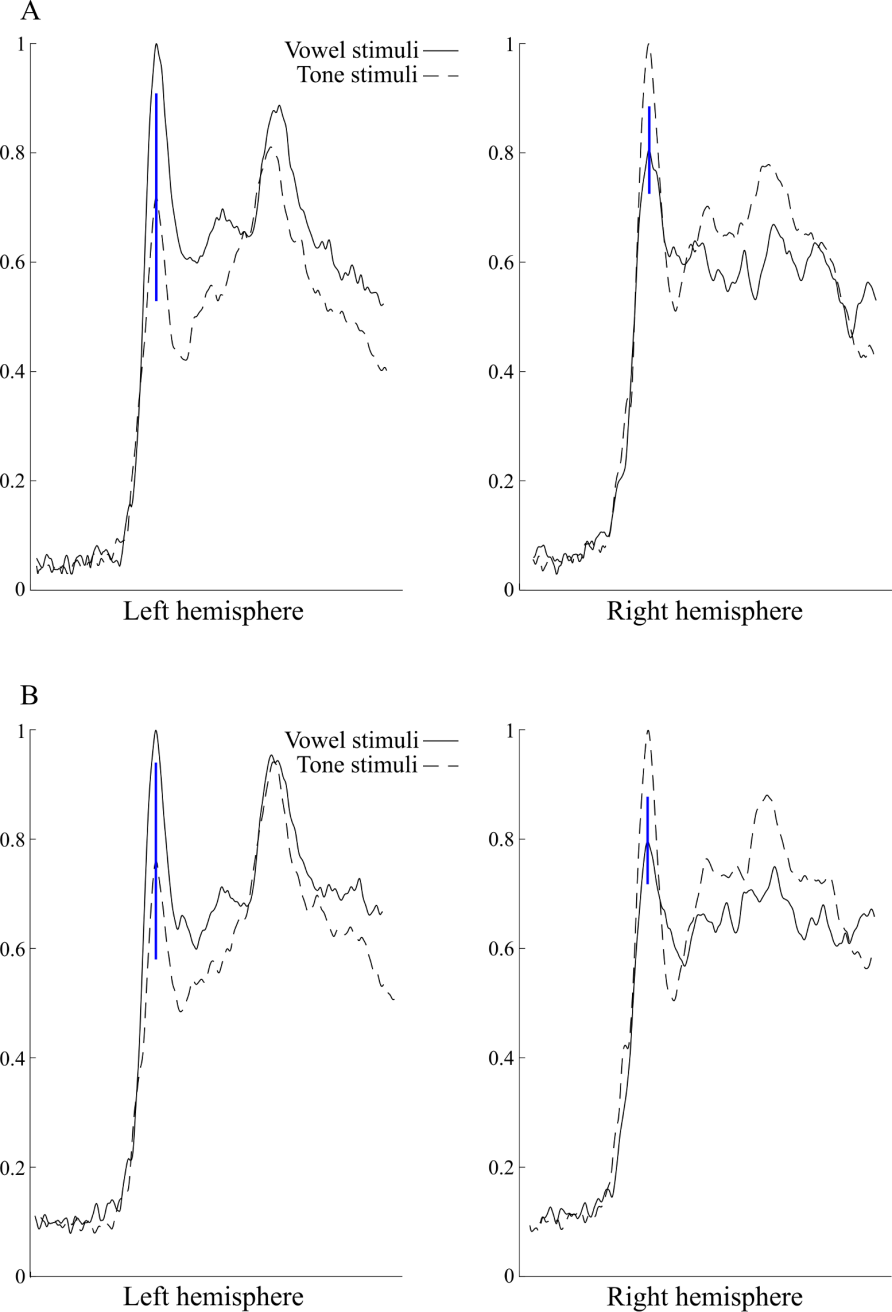
Averaged MEG responses of the patients showing the “left hemisphere language dominant MEG pattern”. Normalized response amplitudes, with the peak amplitude of the stronger N100m response set to the value of one (response to the vowel stimuli in the left hemisphere and response to the tone stimuli in the right hemisphere). The vertical bar marks the 95% confidence interval of the response amplitude. A: Single sensor analysis, B: Regional analysis.

In the analysis based on single gradiometer pair data, six patients showed a pattern of higher peak amplitude of N100m vector sum response to vowel than tone stimuli in the left hemisphere and higher amplitude response to tones than vowels in the right hemisphere; this was defined as the “left hemisphere language dominant MEG pattern” (or “LH MEG pattern”), see Fig 1. All of them had left-hemispheric language dominance based on the IAP. Association between the LH MEG pattern and the left-hemispheric dominance in IAP was statistically significant (Fisher's exact test p = 0.01). Sensitivity of the LH MEG pattern as a predictor of left dominance in the IAP was 67% (6 of 9 patients) and specificity 100%. In the left hemisphere, the N100m response amplitude to tones was on average 72% (95% confidence interval 53–91%) of that to vowels, and in the right hemisphere, the N100m response amplitude to vowels was on average 81% (95% confidence interval 73–89%) of that to tones; see Fig 2A.

When the averaged vector sums of responses over temporal sensors were used for analysis, seven patients displayed the LH MEG pattern. This included all the six patients who showed this pattern in the single sensor analysis. Also in this regional analysis, the LH MEG pattern and the left-hemispheric dominance in IAP were statistically significantly associated (Fisher's exact test p = 0.003). Sensitivity of the regional LH MEG pattern was 78% (7 of 9 patients) and specificity 100%. In the left hemisphere, the regional N100m response amplitude to tones was on average 76% (95% confidence interval 58–94%) of that to vowels, and in the right hemisphere, the regional N100m response amplitude to vowels was on average 80% (95% confidence interval 72–88%) of that to tones; see Fig 2B.

Apart from the LH MEG pattern, no other MEG response pattern was associated with a particular IAP result. Six of the patients displayed a stronger N100m response over both hemispheres to vowel stimuli than to tone stimuli in the single sensor analysis. In the regional analysis, one of these patients had the LH MEG pattern, but the other five had the same pattern as in the single sensor analysis. One patient had stronger responses to tone stimuli than to vowel stimuli in both hemispheres in the single sensor analysis and one additional patient displayed this pattern in the regional analysis. Two patients in the single sensor analysis and one patient in the regional analysis showed stronger response to tone stimuli than to vowel stimuli in the left hemisphere and *vice versa* in the right hemisphere–that is, the reverse of the LH MEG pattern. One patient had no response to tone stimuli in the left hemisphere and a stronger response to vowel stimuli than to tone stimuli in the right hemisphere.

**Fig 3:**
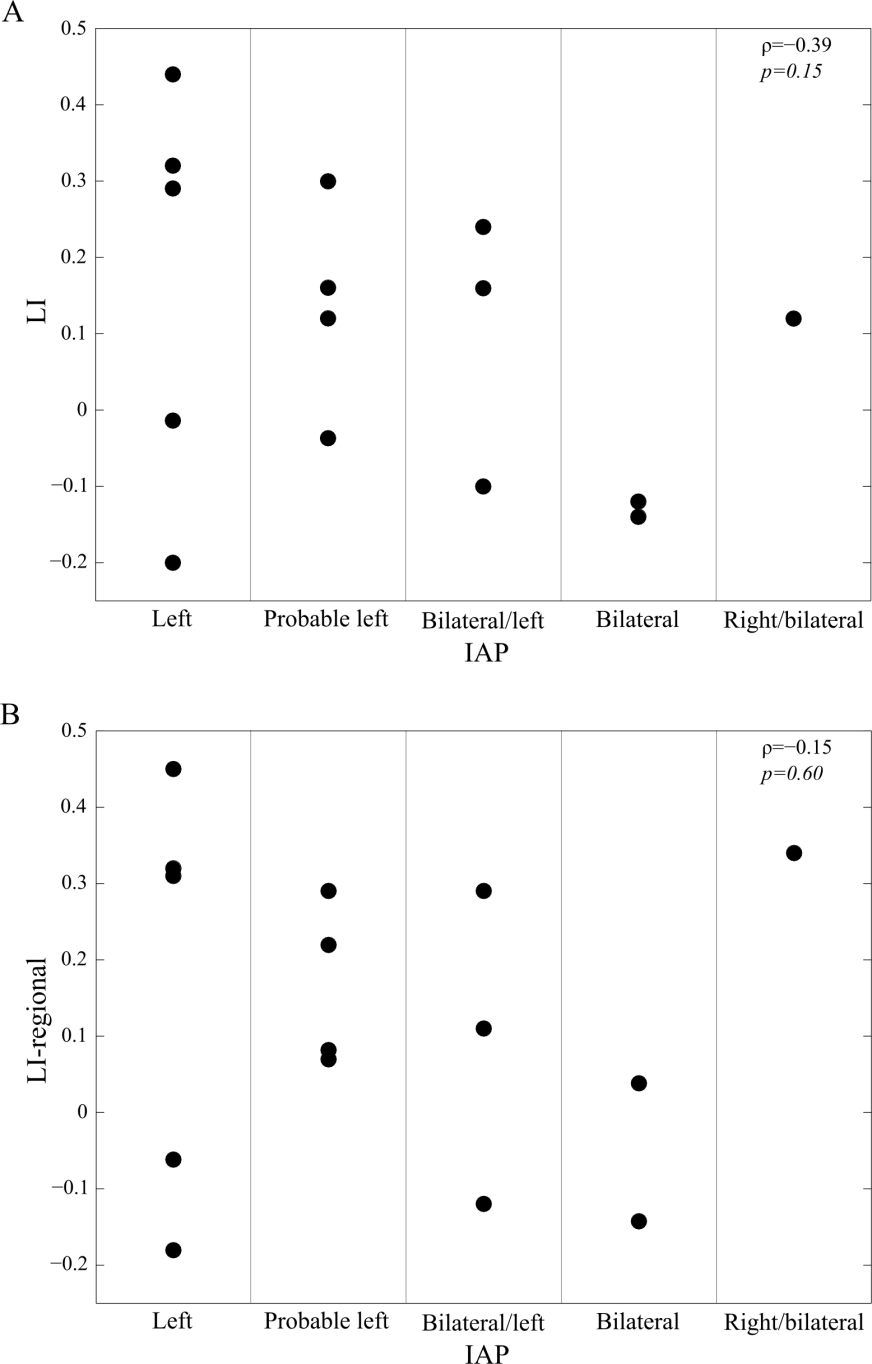
Laterality indices grouped by IAP results. A: LI (single sensor analysis) against IAP. B: LI (regional average analysis) against IAP. ρ is the Spearman's rank correlation coefficient.

The overall correlation between MEG laterality index values and the IAP results categorized as described in Methods was not statistically significant (Spearman rank correlation for single sensor LI data: ρ = –0.39, p = 0.15; and for regional LI data ρ = –0.15, p = 0.60), see Fig 3. There was no significant difference between the laterality indices of the left-dominant patients and those of the non-left-dominant (either bilateral or right-dominant) patients as based on IAP results: median LI (range) was 0.16 (–0.20 to 0.44) for the left-dominant and 0.01 (–0.14 to 0.24) for the non-left-dominant patients (p = 0.22; Wilcoxon rank sum test). Similarly, median LI-regional (range) was 0.22 (–0.18 to 0.45) for the left-dominant and 0.07 (–0.14 to 0.34) for the non-left-dominant patients (p = 0.55). However, there was a trend of a more positive LI in the left-dominant patients than in the non-left-dominant patients.

### Intracranial stimulations

Five of the six patients studied with intracranial electrode stimulations for language functions showed language responses in the left hemisphere. As compared against the IAP, intracranial studies provided additional information on language lateralization in one patient (P12 in Table 2). Stimulation of stereotactic EEG electrodes in the left inferior frontal gyrus (IFG) resulted in an unexpected, yet reproducible, anomia in a picture naming task in this patient. Because of this finding, a unilateral IAP was performed on the left hemisphere and full language function was observed during the test. These findings suggest some degree of bilateral language representation at least for anterior language areas.

### Language outcome after surgery

Five of the 12 patients who had undergone surgery showed a postoperative impairment of language functions. All of these patients either had had an operation on their language-dominant hemisphere or had shown a bilateral language representation, as defined by the IAP. In two of them, language impairment was considered substantial, and in three patients, it was assessed to be mild. One patient showed an improvement of language functions as compared with the preoperative assessment. He was evaluated to be right-dominant in the IAP (although bilateral language functions could not be completely ruled out, because only left-sided injection was performed and direct cortical stimulation by intracranial electrodes in the left hemisphere suggested language function in the left IFG) and was operated on the left hemisphere.

## Discussion

Our results show that a simple auditory MEG paradigm could be used to suggest or to confirm left-hemispheric language dominance in epilepsy patients. We found that although the laterality index calculated from the MEG responses did not correlate significantly with the results of IAP, there was a distinct pattern of MEG responses that was only found in the patients who showed left-hemispheric dominance in the IAP. Furthermore, we found that analyzing a response averaged over multiple temporal channels over both hemispheres instead of a single channel resulted in higher sensitivity of detecting the LH MEG pattern. In visual inspection, the N100m responses were typically quite widespread over the temporal channels prompting us to test the regional average. Notably, the LH MEG pattern was specific to the left hemisphere dominant patients, and although its sensitivity was not perfect, the test succeeded in detecting left-hemispheric language lateralization in most of these patients. Therefore, if the results are confirmed in further studies with larger patient samples, it could eventually be used to reduce the need for the IAP.

Previous MEG language lateralization studies that have analyzed averaged evoked responses in epilepsy patients have mainly concentrated on the analysis of late-onset responses typically appearing at 200 ms or later after the auditory stimulus[13–15]. Our approach was significantly different since we used the early N100m component for our analysis. Any sound stimulus is known to evoke a bilateral superior temporal N100m response in MEG[26]. Speech-related sounds such as words and sentences also evoke a sustained later-onset activation in the superior temporal cortex at approximately 200–800 ms after the stimulus, which is often named the N400m. The N100m response is thought to mainly reflect the acoustic-phonetic characteristics of the stimulus, whereas the more language specific phonetic-phonological and lexical-semantic analysis are reflected by the later-onset responses[27].

However, there is also evidence that the auditory N100m may be modulated by speech-specific contents of the stimulus and that it may show speech-specific lateralization. An MEG study using vowels, consonant-vowel syllables and non-speech sounds as stimuli found that the vowel and consonant-vowel stimuli produced a stronger N100m response than the non-speech sounds in the left hemisphere, but not in the right hemisphere in healthy right-handed subjects[28]. Another MEG study in healthy right-handed subjects showed a similar result when comparing the N100m responses elicited by vowel *vs*. tone or piano sound stimuli[24]. Furthermore, one MEG study reported a phonological priming effect on the N100m response in the left hemisphere in healthy right-handed subjects using spoken words as stimuli[29]

The paradigm we used in this study was previously found to typically elicit stronger N100m responses to vowel *vs*. tone stimuli in both hemispheres in right-handed healthy subjects[20]. The authors reported a larger difference between vowel *vs*. tone response amplitudes in the left than in the right hemisphere. Therefore, the laterality index that was calculated similarly as in the present study, was found to differ between right- and left-handed subjects, suggesting that it could reflect different language lateralizations between these groups.

These previous findings, suggesting that the auditory N100m response contains speech-specific lateralized information, motivated us to investigate its usefulness in studying language lateralization in epilepsy patients. The auditory N100m is a very robust response that is generally easy to elicit and to analyze, except in young children, who were therefore excluded from the present study. All but one of the 16 patients in our study group showed the N100m response to both stimulus categories in both hemispheres. The robustness of the auditory N100m makes it a good candidate for performing partly automatized analysis, which in clinical settings would be an advantage. Additionally, the language task in our test is quite simple to perform and could therefore be useful also in patients with cognitive impairments.

Previous MEG studies have reported highly variable proportions of patients with atypical language lateralization ranging from 9% to 29% as defined by the IAP[13–17]. In our epilepsy surgery program, the IAP is used highly selectively only in the cases where determining language lateralization is considered essential and non-invasive methods have failed. Typically, in these patients the suspected epileptogenic lesion is located near or within the anatomically presumed language areas and they often show clinical symptoms or results from neuropsychological evaluation suggesting the possibility of atypical language lateralization. This explains the unusually high proportion (44%) of patients with atypical language lateralization in our current study.

In the light of previous studies, the determination of typical left-hemispheric language lateralization using noninvasive methods seems more straightforward and reliable than that of atypical lateralization. As mentioned in Introduction, this has been the conclusion of recent fMRI reviews[7,8]. Some previous MEG studies have also shown this phenomenon. Doss et al.[14] reported a higher concordance between MEG and IAP lateralizations in the patients with left dominant IAP than in those with atypical IAP lateralization. They cited the high proportion (29%) of patients with atypical lateralization in their study as one possible explanation for the somewhat lower concordance rate compared to a previous study using the same methodology[13]. The study by Hirata et al.[16] also showed similar difference in the concordance rates (96% *vs* 60%, Fisher’s exact test p<0.01, this analysis is based on the data provided in the original article but performed by the authors of the present study). Similarly, the very high proportion of patients with atypical language lateralization in our study may explain the lack of significant correlation between the MEG LI and the IAP result.

Some epilepsy patients may exhibit discordant lateralization of expressive and receptive language functions based on previous studies utilizing IAP[30], MEG[31] and a combination of fMRI and MEG[32]. It seems reasonable to assume that our MEG paradigm mainly assesses receptive language functions since the task involves no expressive component. Therefore, the comparison with the IAP findings, which represent a combination of both expressive and receptive functions, may be invalid in some patients. This phenomenon may also be one of the reasons why the MEG laterality index values calculated as described in this paper did not show significant correlation with the IAP results.

In conclusion, this simple auditory MEG paradigm shows promise in suggesting or confirming left hemispheric language dominance feasibly and noninvasively in adult and adolescent epilepsy surgery candidates. However, because of our small patient sample, these results can only be considered preliminary. If our results are confirmed in a larger patient population, the test could reduce the need for the invasive IAP. It requires minimal added effort in the patients who would in any case undergo an MEG study to localize epileptic activity. We utilized a simple language task that is quite easy for the patient to perform and could therefore be useful also in patients with cognitive impairments. The data analysis is straightforward and can be partly automatized. The test seems highly specific. While fMRI is currently the most established noninvasive method to study language lateralization, it occasionally fails. In addition, presurgical evaluation of epilepsy surgery candidates always benefits from any confirmatory information gained from multiple independent methods.

## Supporting information

S1 Table Peak latencies, amplitudes and response patterns of the N100m responses. (XLSX)

S2 Table Detection rates of target stimuli. (XLSX)
